# What Happened in ‘The HERizon Project’?—Process Evaluation of a Multi-Arm Remote Physical Activity Intervention for Adolescent Girls

**DOI:** 10.3390/ijerph19020966

**Published:** 2022-01-15

**Authors:** Emma S. Cowley, Lawrence Foweather, Paula M. Watson, Sarahjane Belton, Andrew Thompson, Dick Thijssen, Anton J. M. Wagenmakers

**Affiliations:** 1 Research Institute of Sport and Exercise Science, Liverpool John Moores University, Liverpool L3 3AF, UK; e.s.cowley@ljmu.ac.uk (E.S.C.); l.foweather@ljmu.ac.uk (L.F.); p.m.watson@ljmu.ac.uk (P.M.W.); 2 School of Health and Human Performance, Dublin City University, D09 NA55 Dublin, Ireland; sarahjane.belton@dcu.ie; 3 Wolfson Centre for Personalised Medicine, Institute of Systems, Molecular and Integrative Biology, University of Liverpool, Liverpool L69 3BX, UK; Andrew.thompson@liverpool.ac.uk; 4 Radboud Institute of Health Sciences, Department of Physiology, Radboud University Medical Centre, 6525 GA Nijmegen, The Netherlands; D.Thijssen@ljmu.ac.uk

**Keywords:** process evaluation, remote, intervention, physical activity, adolescence

## Abstract

This mixed-methods process evaluation examines the reach, recruitment, fidelity, adherence, acceptability, mechanisms of impact, and context of remote 12-week physical activity (PA) interventions for adolescent girls named The HERizon Project. The study was comprised of four arms—a PA programme group, a behaviour change support group, a combined group, and a comparison group. Data sources included intervention deliverer and participant logbooks (100 and 71% respective response rates, respectively), exit surveys (72% response rate), and semi-structured focus groups/interviews conducted with a random subsample of participants from each of the intervention arms (*n* = 34). All intervention deliverers received standardised training and successfully completed pre-intervention competency tasks. Based on self-report logs, 99% of mentors adhered to the call guide, and 100% of calls and live workouts were offered. Participant adherence and intervention receipt were also high for all intervention arms. Participants were generally satisfied with the intervention components; however, improvements were recommended for the online social media community within the PA programme and combined intervention arms. Autonomy, sense of accomplishment, accountability, and routine were identified as factors facilitating participant willingness to adhere to the intervention across all intervention arms. Future remote interventions should consider structured group facilitation to encourage a genuine sense of community among participants.

## 1. Introduction

Regular physical activity (PA) is associated with numerous physical [1], psychological [2], cognitive [3], and social [4] benefits for adolescents. Yet, a recent global report found that almost 85% of adolescent girls were not meeting the minimum PA guidelines [5], with moderate-to-vigorous PA (MVPA) declining in girls by 10% annually from the age of 9 years old [6]. The factors influencing adolescent girls’ participation in PA are complex and multifaceted, with common barriers including fear of being judged by others [7], dislike of PA [8], and a perception that femininity is incompatible with being physically active [9,10].

In response to low PA participation, there has been an increase in interventions specifically targeting adolescent girls [11,12,13]. Due to the inherent difficulty in changing an individual’s health behaviours [14], many PA interventions implement several interacting components, for example providing informational material, mentorship, group support, and reward structures [15,16,17,18]. Further, PA programmes are often set in schools and communities which exist within complex systems, where there are multiple contextual factors, often that cannot be controlled or accounted for by researchers [19,20]. Due to this complexity, understanding how PA interventions are implemented is crucial to having confidence in their effectiveness [21].

Traditionally, much of the focus in randomised controlled trials was placed on the effectiveness of an intervention, with little priority given to understanding why an intervention was or was not successful [22]. By conducting a comprehensive process evaluation to understand if an intervention has been delivered as intended, the internal and external validity can be improved, allowing the intervention to be replicated and applied to real-world settings [23]. In this way, researchers can have greater confidence in an intervention’s outcomes, as without investigating programme design and implementation, incorrect conclusions can be made about an intervention’s effectiveness. For example, an intervention may be discarded due to nonsignificant results, however it is not known if the intervention itself is ineffective, or if null findings are due to some unknown factor [24]. Further uses of process evaluation include assessing if the target audience has been recruited, how much of the intervention was delivered and received, refining the intervention to enhance its appropriateness, and scaling it up for larger populations or different settings [21].

Process evaluations are often guided by several frameworks [21,23,25,26,27,28,29], and although there are commonalities across many frameworks (e.g., reach, dose, fidelity, and context), there is no consensus on what components should be included in the evaluation, nor agreed upon definitions for these components. This often makes it difficult to implement the findings of process evaluations into practice as the terms used can have different meanings. The present article reports a process evaluation of an adolescent girls PA intervention study called The HERizon Project (described below). Using the data sources available, the process evaluation draws on elements from several frameworks, specifically: (i) *reach and recruitment*, how representative participants are of the target population and the methods used to approach participants [28]; (ii) *fidelity of delivery*, the degree to which interventions have been implemented as intended [26]; (iii) *participant receipt, engagement, and enactment*, the amount of the intervention received by participants, and the extent to which they understand the key components and can put this knowledge to use in everyday life [23]; (iv) *adherence*, participant’s compliance to the intervention’s prescribed treatment [23]; (v) *acceptability*, stakeholders perceived appropriateness and satisfaction of the intervention [29]; (vi) *mechanisms of impact*, understanding the ways in which the intervention brings about change [21]; and (vii) *context,* consideration of any external factors that influence the implementation of an intervention [21].

‘The HERizon Project’ was a randomised controlled trial (RCT) which aimed to evaluate the effect of remote PA interventions designed to increase PA levels of adolescent girls living in the UK and Ireland. Following on from previous formative work [7], the trial consisted of four arms: PA programme group, behaviour change support group, combined PA programme group and behaviour change support group, and a comparison group (Table 1). This mixed-method process evaluation aims to report on what components, and their dose, were implemented in each intervention arm, which factors influenced the trial recruitment and implementation, and participants’ perceived acceptability and enjoyment. To reduce biased interpretation of data, this process evaluation of The HERizon Project was conducted prior to outcome analysis [30]. The results will provide prospective insights into the interventions effectiveness, and reasoning for its success or non-success [21].

## 2. Materials and Methods

### 2.1. Study Design

Ethical approval for The HERizon Project was obtained from Liverpool John Moores Research Ethics Committee (20/SPS/042) and the study was registered with clinicaltrials.gov (reference: NCT04766372). The HERizon Project aimed to increase the PA levels of adolescent girls in the UK and Ireland. Knowledge gained from earlier formative work and a feasibility study was instrumental in the design of the current study [7,10]. This RCT assessed whether three intervention arms: (i) PA programme group, (ii) behaviour change support group, (iii) combined PA programme and behaviour change support group, each delivered remotely for 12 weeks, increased MVPA compared to a (iv) comparison group. Due to the nature of the intervention, it was not possible to blind participants or intervention deliverers. Data were collected remotely at baseline (T0—December 2020 to January 2021), postintervention (T1—March to April 2021), and 3 months following the end of the intervention (T2—July to August 2021). After baseline measurements, participants were block randomised with country-level (UK and Ireland) stratification using Microsoft Excel (Version 16 for Mac, Microsoft Corporation, Washington, DC, USA). Girls who enrolled with a sister/friend/classmate were considered a cluster and were therefore cluster-randomised into the same intervention arm to minimise contamination. The primary outcome was objective MVPA, measured by 9-day wrist-worn accelerometer (GT9X and GT3X+ models, Actigraph, Florida, FL, USA). Secondary outcomes included cardiorespiratory fitness, muscular strength and endurance, exercise motivation, perceived competence, self-esteem, and body appreciation. Intervention outcomes will be reported elsewhere. The present study focuses on the mixed-methods process evaluation.

### 2.2. Participants and Recruitment

Based on the median sample sizes of feasibility trials within the UK Clinical Research Network database [31], The HERizon Project feasibility trial aimed to recruit 160 participants, with equal distribution between study arms. Girls living in the UK or Ireland, aged between 13–16 years old, who wished for support in increasing their PA were eligible for inclusion. Exclusion criteria were: (a) a condition that prevented them from engaging in moderate intensity PA, (b) pregnancy, and (c) not having access to a smartphone or computer. All participants and their parents/guardians provided written informed assent/consent prior to baseline measurements. Social media advertisements, and links with local school and community groups, were used to recruit participants.

### 2.3. Interventions

This study involved four groups: PA programme group, behaviour change support group, combined group, and a comparison group. Participants in the comparison and all intervention arms were asked to complete three 30-min PA sessions of their choosing each week for the duration of the intervention. All participants were sent a hardcopy 25-page PA logbook to their home address at the beginning of the 12-week intervention, as well as a digital copy to their nominated email address (Supplementary Figure S1). The logbook contained a range of suggested PA options that could be conducted at home during COVID-19 restrictions, e.g., YouTube and Instagram video links to follow-along dance, Pilates, yoga, boxing, and resistance training workouts. The logbook also contained optional worksheets on various topics that participants were invited to complete.

#### 2.3.1. PA Programme Group

Participants allocated to the PA programme group received three standardised no-reply text messages each week which provided PA reminders (e.g., “Reminder—live workouts this week are Wednesday at 6.30 pm and Saturday at 10 am.”), encouragement (“Try not to get overwhelmed, remember that small steps lead to big changes!”), and support (e.g., “If you have any questions please send us an email to (researcher email address)). An online text message service was used to schedule and send text messages each week on the same day/time. Participants also had access to two live group workouts each week led by the lead author (a certified personal trainer with experience leading group exercise classes). Each session lasted approximately 40 min and consisted of a dynamic warm-up, followed by a series of bodyweight exercises including squats, push ups, lunges, and cardiovascular exercises, and concluded with static stretching. To cater for participants who could not attend the live workouts, all sessions were recorded and uploaded to an online folder which allowed participants to take part in classes at a later time, or to repeat workouts that they particularly enjoyed. Participants were also invited to join a private Instagram group chat, moderated by a researcher, where they could communicate with other girls from their intervention arm.

#### 2.3.2. Behaviour Change Support Group

Participants in the behaviour change support group were paired with an ‘Activity Mentor’ whom they worked with for the duration of the intervention. All Activity Mentors (*n* = 12) were Master of Science (MSc) and professional doctorate trainee sport and exercise psychologists and were supervised by a Health and Care Professions Council registered sport and exercise psychologist (third author). To increase the likelihood of participants receiving similar behaviour change support, a standardised approach was used to recruit and train Activity Mentors. Prospective mentors submitted an application form and were interviewed by the first and third authors, using a pre-prepared question guide. Activity Mentors were hired based on their experience, qualifications, and characteristics. All Activity Mentors were asked to complete virtual training led by the third author and all resources used within the workshops were made available to mentors for future reference. Prior to the training, Activity Mentors received a 50-page intervention manual which included detailed information regarding needs-supportive delivery, as well as process and procedure documents to ensure arising issues were dealt with in a consistent manner, e.g., should safeguarding concerns arise and how to follow-up with no-shows. Activity Mentors engaged in interactive competency tasks, such as roleplay, which were reviewed by a senior mentor who provided constructive feedback. Activity Mentors were assigned to participants based on matching availability and participants were then given a weekly time slot for when subsequent calls would occur. Introduction and week 12 videocalls were scheduled to last 30 min, with videocalls on weeks 1–6 and 9 scheduled to last approximately 15 min. To standardise videocalls, a pre-planned session guide was employed (Supplementary Table S1). Sessions drew on self-determination theory [32] and focused on fostering participant autonomy, competence, and relatedness through the use of motivational and behaviour change techniques.

#### 2.3.3. Combined PA Programme and Behaviour Change Support Group

Participants in the combined group received all intervention components, i.e., PA logbook, three standardised no-reply text messages each week, access to two live group workouts each week, and to a private Instagram group chat and were partnered with an Activity Mentor for videocalls on weeks 0, 1–6, 9, and 12.

#### 2.3.4. Comparison Group

Participants in the comparison group received only the PA logbook component and no additional contact from the research team outside of data collection time points.

### 2.4. Process Evaluation Framework and Data Collection

This mixed-methods process evaluation used a modified framework [21,23,25,26,27,28,29]. Our adapted framework specifically explored reach and recruitment, delivery fidelity, participant receipt and enactment, adherence, acceptability, mechanisms of impact, and context (see Table 2).

### 2.5. Data Sources

#### 2.5.1. Demographic Data

Participants’ age, country, menstruation status, ethnicity, and home postcode were collected via an online form at baseline. The last three digits of participants home postcodes were used to estimate socioeconomic status by mapping against indices of multiple deprivation [33,34].

#### 2.5.2. PA Logbooks

Using the logbook, participants were asked to record their PA each week, including details of the day they were active and the type of PA they did. Participants in the behaviour change support and combined groups discussed their logbook with their Activity Mentor during weekly videocalls and talked through the corresponding topic, e.g., mentors supported participants in setting goals and developing strategies to overcome barriers that may stop them from reaching their goals.

#### 2.5.3. Activity Mentor Logbooks

Activity Mentors kept a weekly logbook for each participant they had been assigned, which included a record of call attendance (yes/no), call duration (minutes), number of sessions and type of PA completed by each participant (e.g., 3 PA sessions—jog, live workout, and hike), and whether the session was delivered in accordance with pre-planned session guide (yes/no).

#### 2.5.4. Exit Surveys

At the end of the intervention, all participants were asked to complete an anonymous online exit survey which gathered opinions on intervention content, delivery, perceived choice, perceived impact of the intervention, and participants most/least favourite thing about taking part in HERizon (Supplementary Table S2). Surveys contained a mix of open and closed questions and were tailored according to the intervention arm. Closed questions were scored using a 5-point Likert scale ranging from “Not at all” (1) to “Very much” (5). As a measure of engagement, participants from all intervention arms were asked how much they used the logbook to record their PA sessions, and if they used any of the suggested physical activities. To quantitatively assess participant comprehension and enactment of intervention components into their daily life, surveys asked participants “do you feel you understand the reasons for being physically active and their importance?” and “has The HERizon Project helped you improve your attitudes/behaviours towards PA?”. Questions regarding local COVID-19 restrictions were also identical across all surveys, e.g., “were non-essential shops open? E.g., retail stores”, however questions that related to specific intervention components (e.g., behaviour change support calls, live workouts, text messages, and online Instagram groups) differed by intervention arm.

#### 2.5.5. Focus Groups and Interviews

Semi-structured focus groups and interviews took place with a randomly selected sub-sample of participants allocated to PA programme (*n* = 11), behaviour change support (*n* = 11), and combined groups (*n* = 12) after postintervention data collection. Participants were selected using the random number generator tool on Microsoft Excel (Version 16 for Mac, Microsoft Corporation, Washington, USA). All focus groups and interviews were conducted online using Microsoft Teams and lasted between 20 to 45 min. A pre-planned interview schedule, which consisted of open and closed questions, was used to facilitate discussion around intervention recruitment, delivery, perceived impact, and future recommendations (Supplementary Table S3). Focus groups and interviews were conducted by the first author.

### 2.6. Data Analyses

Process evaluation findings are presented using both qualitative and quantitative data sources. Response rates and time points are outlined in Table 3.

#### 2.6.1. Analysis of Quantitative Data

Data on demographics, attrition, delivery fidelity, adherence, and quantitative responses to exit survey questions were analysed using descriptive statistics using Microsoft Excel and are presented as the mean ± SD, unless otherwise stated. Analysis of variance (ANCOVA) and independent *t*-tests were conducted to investigate significant differences between groups. Statistical analysis was performed using SPSS for Mac (version 27, SPSS, Chicago, IL, USA), with a *p*-value of 0.05 used to denote statistical significance.

#### 2.6.2. Analysis of Qualitative Data

Responses to open ended exit survey questions were recorded in Microsoft Excel and recurrent points were grouped into themes. Focus groups and interview transcripts were uploaded to NVivo 12 (QRS International, Doncaster, Australia), and, following data familiarisation, themes were identified using reflective thematic analysis [35]. Initially, themes were identified in a deductive manner, using a-priori process evaluation questions as a start point, following which an inductive approach was used to identify any further themes. The initial thematic structure was developed by the first author. To enhance rigour and ensure alternative perspectives of data were considered, sections of raw data were reviewed by the second, third, and fourth authors [36]. Due to the large volume of data collected, participant quotes that were considered most informative and important are used within the results section. Quotes are used to illustrate the process evaluation component being discussed and to provide richer meaning and context to the quantitative outcomes.

## 3. Results

### 3.1. Participant Descriptives

**Figure 1:**
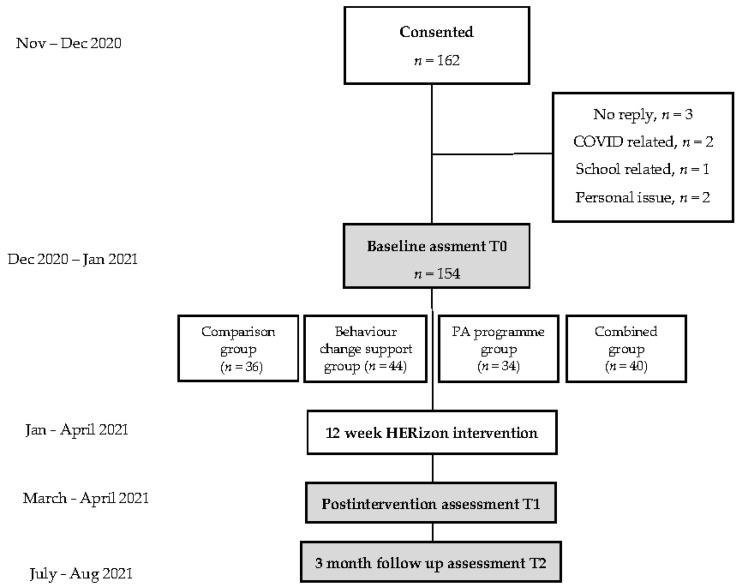
Overview of the HERizon study design.

In total, 189 participants expressed an interest in taking part in the study, of which 162 provided written informed consent (86% recruitment rate). As shown in Figure 1, the baseline measures were collected from 154 participants, and 111 participants completed all or some follow-up measures (69% overall response rate). Reasons for drop out from consent to baseline assessment included unrelated injuries (*n* = 1), school stress (*n* = 3), and personal issues (*n* = 4). Descriptive characteristics are presented in Table 4. There were no significant differences in demographics or PA habits between groups.

Based on focus group and interview data, the use of paid advertisements on social media was the most successful recruitment method in the trial, with the majority of girls citing Instagram as the location where they found information on HERizon. Girls reported that the most common reasons for signing up to the study were, (i) boredom during COVID-19 lockdown restrictions, (ii) to become physically active and improve health habits, and (iii) felt motivated by the commitment of 12 weeks and access to an online community.

### 3.2. Reach and Recruitment

#### 3.2.1. PA Programme Group

The mean age of girls in the PA programme group was 15.3 years, and the majority of participants were white (81%). A total of 58% of participants lived in the UK, with over half of all the group’s participants living in areas within the median deprivation tertile (56%). According to baseline self-report PA questionnaire, girls were physically active for 60 min for on average two days per week, and no participants met the government guidelines of 60 min of daily MVPA.

#### 3.2.2. Behaviour Change Support Group

Girls in the behaviour change support group had a mean age of 14.6 years, and similar to the PA programme group, the majority were white (77%). A total of 46% of girls lived in the UK and a large proportion lived in areas of median deprivation (45%), with an even proportion living in the most (20%) and least (20%) deprivation tertiles. Girls in this group reported, via baseline self-report PA questionnaires, to engage in the same amount of PA as girls in the PA programme group: 60 min for on average two days per week, and no participants met the government guidelines of 60 min of daily MVPA.

#### 3.2.3. Combined Group

The mean age of girls in the combined group was 14.9 years, with 56% living in the UK. Similar to previous groups, the majority of participants were white (82%) and lived in the areas within the median deprivation tertile (68%). No participants met the government PA guidelines, and the mean number of days girls were physically active for 60 min was 2.5 days.

### 3.3. Delivery Fidelity

#### 3.3.1. PA Programme Group

PA logbooks were sent to all participants in the PA programme group (*n* = 36) and all participants were invited to join the private Instagram group. Over the course of the twelve-week intervention, three text messages were sent each week (*n* = 1294). All planned live group workouts were delivered as intended on Wednesday evenings (*n* = 12) and Saturday mornings (*n* = 12). The minimum length of live group workouts was 30.1 min, maximum length was 37.4 min, and the average length was 34.1 min.

#### 3.3.2. Behaviour Change Support Group

PA logbooks were sent to all participants in the behaviour change support group (*n* = 44). Based on Activity Mentors logbooks, all nine videocalls were scheduled for each participant and mentors reported 100% adherence to the pre-planned call guide. One mentor, when asked about the key challenges of implementing the intervention, commented:

‘*My main challenge was that some participants missed a number of their scheduled sessions. On my part this meant attempting to reschedule at a mutually convenient time, but sometimes it was hard work to even get a response from the girls!*’

#### 3.3.3. Combined PA Programme and Behaviour Change Support Group

Similar to previous groups, all participants were sent a PA logbook (*n* = 34), were invited to join the private Instagram group, and all Activity Mentor videocalls were scheduled for each participant, with mentors adhering to 99% of the pre-planned call guide. Three text messages were sent to each participant every week for the duration of the intervention (*n* = 1224). All planned live group workouts were delivered as intended on Wednesday evenings (*n* = 12) and Saturday mornings (*n* = 12). Some participants could not attend these sessions as timings were inconvenient. When asked about the delivery of live group workouts, one participant said:

‘*I work Saturdays so the Saturday morning never worked for me… so I would go back the following day into the (online folder) link and do the workouts then… I liked the flexibility*’

(Combination group, 16, Ireland)

### 3.4. Participant Receipt, Engagement, and Enactment

#### 3.4.1. PA Programme Group

All participants received a PA logbook (*n* = 36) and 81% of participants reported using the logbook to record their weekly PA sessions. During focus groups, many participants said they used the suggested PA resources in the logbook and enjoyed trying different types of exercise:

‘*I tried a few of the videos and I liked some more than others but because you have such a variety it is really good because different people can try lots of different things*’

(PA programme group, 14, UK)

In total, 71% of participants reported that they took part in at least one live group workout, 24 participants joined the private Instagram group (70.5%), and 97% of text messages were delivered (all text messages to one participant failed to successfully deliver due to an incorrect phone number). Many of the girls spoke of finding the text messages to be good reminders to fill in their logbooks, as well as being sources of encouragement to complete their PA sessions:

‘*I loved (the text messages) because they reminded me to fill in the logbook. They were also quite motivational to keep going like if you thought “I don’t want to do this exercise today” so that was nice*’

(PA programme group, 15, Ireland)

Exit surveys revealed that PA programme participants’ understanding of the reasons for being active were high (4.7 ± 0.7 out of 5). Further, participants reported a large improvement in attitudes and behaviours towards PA following the HERizon intervention (4.2 ± 0.8 out of 5), with one participant reporting a positive change in how she views PA:

‘*My outlook on exercise has definitely changed I used to look at exercise as a chore but now it’s something that I wake up and I really want to do so (HERizon) has definitely affected my mindset*’

(PA programme group, 15, Ireland)

#### 3.4.2. Behaviour Change Support Group

All participants received a PA logbook (*n* = 44) and 76% reported they used their logbooks to report their weekly PA. All participants were partnered with an Activity Mentor and the mean number of videocalls participants attended was 7 (±2). Introduction videocalls lasted 30 (±6.6) min, calls on weeks 1–6 and 9 lasted 13 (±3.4) min, and the final call on week 12 lasted 23 (±7.6) min. The total mean call duration was 134 (±40.5) min per participant. Similar to the PA programme group, based on exit surveys, participants had a high understanding of the importance of being physically active (4.7 ± 0.6 out of 5) and many saw improvements in their attitudes and behaviours towards being PA as a result of taking part in HERizon (4.4 ± 0.9 out of 5), with one participant commenting:

‘*I have got more confident now especially in PE in school because I used to not be very confident and really self-conscious but now I am like “oh ye I can do exercise”*’

(behaviour change group, 14, UK)

#### 3.4.3. Combined PA Programme and Behaviour Change Support Group

All participants received a PA logbook (*n* = 34) and similar to the previous groups, a large proportion of participants (79%) reported they used the logbook to record their weekly PA sessions.

‘*I thought (the logbook) was really useful, I found motivation from it and support and it helped me plan out the week like I got better at planning my exercise and it was nice to look back at it to see how much exercise you’ve done*’

(behaviour change support group, 16, Ireland)

A total of 99.5% of non-reply text messages were delivered (text messages from weeks 1 to 6 for one participant failed to successfully deliver due to an incorrect phone number) and, similarly to the PA programme group, 71% of participants said they tried at least one live group workout. Thirty participants joined the private Instagram group (88%), however the majority of participants said during focus groups that they did not use the group much as they either did not use Instagram at all, or they did not feel comfortable putting messages into the group:

‘*A lot of the time the group chat was just silent unless (the researcher) sent things in… our group didn’t really talk but I’m not really sure how to fix that*’

(combined group, 15, Ireland)

All participants were partnered with an Activity Mentor and the mean number of videocalls participants attended was 8 (±1). Introduction videocalls lasted 28 (±5.3) min, calls on weeks 1–6 and 9 lasted 13 (±2.3) min and the final call on week 12 lasted 21 (±6.6) min. The total mean call duration was 138 (±22.1) min per participant. Reflecting scores of the previous groups, exit surveys revealed participants in the combined group had high understanding of the reasons for being physically active (4.5 ± 0.7 out of 5), and positive improvements in attitudes and behaviours towards PA (3.9 ± 1.1 out of 5). During focus groups, one participant commented on her increased determination during school physical education (PE):

‘*I have such positivity now around exercise cause I never liked PE cause we do the same thing all the time and I’m not very sporty I have no coordination but last Thursday we were doing laps and before I would have given up but I was thinking “I can do it” I felt determination, motivation, and body positivity as well*’

(combined group, 16, Ireland)

### 3.5. Adherence

#### 3.5.1. PA Programme Group

The average number of PA sessions completed by participants in the PA programme group was 34 (±4), with 78% of participants completing three PA sessions per week for 12 weeks). During focus groups, most girls said that three PA sessions per week was an achievable goal, even for those who were not active before the intervention:

‘*I think (three PA sessions) was perfect because it’s not too much especially when you first start (exercising) it can be tough but with 3 it’s easy, it is a small goal, like very achievable*’

(PA programme group, 14, UK)

#### 3.5.2. Behaviour Change Support Group

The average number of PA sessions completed by participants in the behaviour change group throughout the intervention was 32 (±8), with 39% of participants completing three sessions per week for 12 weeks. During focus groups, some participants commented that initially they found three PA sessions per week difficult but over time it got easier as PA became part of their routine:

‘*At the start I really didn’t want to do (PA) at all and I thought about dropping out but then I started actually enjoying it and I found that three times wasn’t enough and wanted to do (PA) four times*’

(behaviour change support group, 16, UK)

#### 3.5.3. Combined PA Programme and Behaviour Change Support Group

The average number of PA sessions completed by participants in the combined group was 33 (±6), with 47% of participants completing three sessions per week for 12 weeks. Similar to the behaviour change support group, girls in the combined group spoke of needing a couple of weeks to settle into the programme and their new routines:

‘*On the 1st week I only did 2 (PA sessions) because I think we were all only getting into (the programme) and used to it but then I stuck to 3 sessions a week*’

(combined group, 15, Ireland)

### 3.6. Acceptability

#### 3.6.1. PA Programme Group

Participant’s perspectives of live group workouts and text messages were mainly positive according to exit survey responses (89% of participants would recommend the HERizon programme to a friend, 87% enjoyed live group workouts, and all participants reported the frequency of non-reply text messages to be ‘just right’). This was further supported by focus group and interview data:

‘*I really liked the live classes like honestly I would pay for it and do them forever I really enjoyed the layout of them and how motivating it was not having to repeat the exercises and I enjoyed that you told us what part of the body you should be working that helped me really focus*’

(PA programme group, 16, UK)

The private Instagram group scored the lowest as when participants were asked to rate their enjoyment of the group on a 5-point Likert scale (1—I did not enjoy it at all, 5—I really enjoyed it) the mean score was 3.31 (±0.6) out of 5. During focus groups, participants spoke of not feeling comfortable initiating conversation in the group as they did not know the other participants:

‘*I just felt like I was scared to speak there because no one was speaking… maybe more ice breakers could be a nice thing like questions (posed by the researcher) for next time*’

(PA programme group,15, Ireland)

#### 3.6.2. Behaviour Change Support Group

The PA logbook was positively received by participants in the behaviour change support group. During focus groups, a number of participants said they found the logbook to be a tool for self-reflection:

‘*I thought (the logbook) was really useful, I found motivation from it and support and it helped me plan out the week like I got better at it, it was nice to look back at it to see how much exercise you’ve done*’

(behaviour change support group, 16, Ireland)

Participants reported high ratings when asked how comfortable they felt talking to their mentor (mean score of 4.47 out of 5, 1—not comfortable at all, 5—very comfortable), 87% of participants reported call length as being “just right”, and 94% said they would want a mentor again if they took part in another HERizon programme. Responses to exit surveys found that participants felt they could be honest with their mentor as they believed they would not be punished if they did not manage to complete all three PA sessions for that week, with one participant commenting, “Calls made me feel better if I missed a (PA) session”. Participants also spoke of enjoying the weekly calls because it was an opportunity to speak with someone new. They said they enjoyed forming a relationship with someone outside of their family or school friend group and that they looked forward to the calls because they felt they were catching up with a friend. Another participant said her favourite part of having an Activity Mentor was having a space each week “to talk to someone about exercise and it not feel like a competition”. In focus groups, many of the participants felt the weekly calls acted as a source of accountability, with one participant commenting:

‘*You felt like you kind of had to do (PA) because someone else was involved like you had to report back and you didn’t want to be like “no I didn’t do anything”*’

(behaviour change support group, 16, UK)

Mentor calls were weekly for the first six weeks of the intervention and then moved to once every three weeks for the remainder of the programme. Although participants generally spoke of enjoying the tapered support, as it gave them a sense of accomplishment when they completed their PA sessions without external monitoring, many struggled with the first break in calls as they found the removal of support too abrupt. During focus groups, participants suggested a more gradual weaning of support would be helpful, e.g., fortnightly calls or a text message in lieu of the weekly call:

‘*In the first break a text would have been beneficial but the second break it good to start building your routine because that is when I found my routine really started in the second break*’

(behaviour change support group, 16, Ireland)

#### 3.6.3. Combined PA Programme and Behaviour Change Support Group

Based on exit surveys responses, acceptability for intervention components was high for the combined group (97% would recommend HERizon to a friend, 94% were satisfied with the amount of help they received during Activity Mentor calls, and 92% enjoyed live group workouts). Similar to the PA programme group, enjoyment of the Instagram group scored the lowest (3.43 ± 1.3 out of 5), with many girls echoing similar viewpoints to those in the PA programme group in that they found it difficult to communicate easily with others in their intervention arm:

‘*I think that the Instagram group was good but it is kind of hard to communicate through it but it was nice just to know that it was there so you could see that there are other people doing the project with you that you’re not alone in it*’

(combined group, 14, Ireland)

Participants felt they had many PA options within the programme and exit surveys responses revealed high scores for perceived choice and autonomy (4.55 ± 0.7 out of 5). One participant in the combined group said she enjoyed the suggested PA options because she was able to try “so many different workouts that I wouldn’t have ever done before”.

### 3.7. Mechanisms of Impact

#### 3.7.1. Routine

Across all intervention arms, many participants showed an awareness of their procrastination around PA. Participants spoke of finding the rigidity of a set timetable for live group workouts particularly helpful, as they felt it made them commit to doing PA on a set day and time:

‘*I really liked the schedule of the workouts because even if I am tired I would be like “OK I am just going to do this and then I can be finished” so I wasn’t like dilly daddling, it was at a set time I couldn’t be like “oh I’ll just do it in another hour”*’

(PA programme group, 15 Ireland)

One participant attended both live group workouts each week but did not do any other PA. When asked about this during an interview she said she would have completed three PA sessions had there have been a third live workout as she said she “feels like when I am pressured with time I do better”.

A participant of the combined group said during focus groups that PA had become part of her weekly routine and therefore being physically active required less effort:

‘*I’m not as afraid of just starting doing physical activity now like it is part of my life but it’s not too big of a part it is just balanced and ye it’s just easy now*’

(combined group, 15, Ireland)

#### 3.7.2. Sense of Accomplishment

There was a strong sense of accomplishment evident within the data at the end of the 12 weeks in all intervention arms. When participants were asked what their favourite part of the programme was, one participant in the behaviour change support group responded “my favourite part of HERizon was achieving goals I set myself’. Another participant found looking back on the PA sessions that she had completed in her logbook to be a great source of motivation:

‘*It gave me a big sense of accomplishment like looking over it and if I did quite a lot (of PA) that week it was good to be able to see what I had done each day… it gave me kind of motivation*’

(behaviour change support group, 15, UK)

Further, participants in the combined group expressed excitement regarding their accomplishments over the course of the intervention, with one participant saying:

‘*I definitely think I have become physically fitter I am definitely stronger like the first strength workout I did I was so sore I could hardly lift the little tin cans over my head but now it is really cool because now I can feel on my arms there is muscle definition*’

(combined group, 16, Ireland)

#### 3.7.3. Accountability

Participants who were partnered with an Activity Mentor (behaviour change support and combined groups) spoke about their weekly videocalls being a source of accountability. Knowing they would speak to their mentor, and talk through the PA logbook, motivated participants to complete their three PA sessions:

‘*I feel like since we had the mentor calls and we knew it had to happen every week it kind of motivated me to do more so I could tell her and not just be like “ye I sat down all day*”.

(combined group, 14, UK)

Participants in the PA programme group commented on their PA logbook being a source of motivation and accountability. Many of the participants said they would plan their PA sessions out for the coming week and used the optional worksheets to reflect on their goals:

‘*I really liked the bit where you could plan (PA) out, it just makes it a lot easier to plan out what you’re going to do for the week and hold yourself accountable, as well the little bit like the week by week topics I quite enjoyed that it was nice to check up on myself*’

(PA programme group, 16, UK)

However, not all participants found the logbook useful. Some participants did not use any of the suggested PA options, and others created their own PA calendar using Google Docs (Google, Mountain View, United States) and the Notion mobile application (Notion API, San Francisco, United States). One participant said she did not use the logbook for anything other than recording attendance at live workouts: ‘I didn’t end up trying anything from the logbook, it just stayed in one position, I would just write in the live workout and then that logbook would be closed and far away from me’ (PA programme group, 16, UK).

#### 3.7.4. Discovery of Preferred PA

Following the programme, many of the participants commented that their perception of what constitutes PA had changed, and that since finding an activity that they enjoy, they have become more physically active in their day to day life:

‘*I am a lot more physically active now… I used to look at exercise like “Why would someone do that? That looks too hard and too boring”, but now I really enjoy it because of all the options we had at the start and the different (physical activities) you can do*’

(behaviour change support group, 13, UK)

Further, as the programme progressed, it seems that PA became less of a task that needed to be done specifically for a research study, and more of something that was part of their everyday life:

‘*It was more routine than feeling like I had to get up and do the project, it kind of felt like you were just doing your thing like on Monday’s I just knew that I had a workout to do, it was routine, like motivation wasn’t needed as much*’

(behaviour change support group, 16, Ireland)

### 3.8. Context

Given the complex nature of the intervention, due to its multiple components, different geographic locations, real-world setting, and implementation during the COVID-19 pandemic, it was deemed important to consider the influence of the broader social and physical environments in which the intervention was carried out. Responses from exit surveys found no significant differences between intervention arms and therefore results are reported at the trial level. Overall, 91% of participants remained in strict COVID-19 lockdown restrictions during post intervention data collection (closure of non-essential retail and indoor restaurant dining, travel restrictions, and prohibition of indoor social gatherings outside of an individual’s “social bubbles”), with 78% of participants returning to in-person schooling from March 2021 (week 8 of the intervention). A total of 59% of participants had no physical education (PE) during COVID-19 school closures. Of those who had remote PE, the main activities were teacher-led Zoom classes or self-led YouTube workout videos, with total PE time lasting on average 60 min to 120 min per week.

A large proportion of participants (75%) reported their weekly commitments had changed since returning to school, with the main commitments being an increase in schoolwork, extracurricular activities, and part-time employment. Sixty-four percent of participants also reported a change in their behaviour and motivation towards PA since returning to school with the re-opening of sports clubs being a key motivator, and lack of time and increased stress listed as the primary drivers of lower motivation.

## 4. Discussion

To the best of the authors knowledge, this investigation is the first multi-arm process evaluation of a PA intervention, and in order to address specific research questions, it was important to develop a bespoke process evaluation framework using elements of previously established frameworks [21,23,25,26,27,28,29]. This ensured the evaluation did not become a ‘tick box’ exercise and used the available data in the most meaningful and informative ways. Overall, results indicate that trial recruitment strategies were successful (86% recruitment rate), implementation fidelity was high, as was intervention adherence. The majority of intervention components were positively received by participants, however the private Instagram group chat was weakly implemented, and had the least satisfaction and perceived use of all intervention components.

Findings suggest that the remote nature and flexibility of the intervention were important facilitators in its high implementation fidelity and adherence. Although the intervention commenced during COVID-19 lockdown restrictions, participants from all intervention arms commented during focus groups and in exit surveys that they liked the programme being online, separate from school, and recommended it stay in an online format, even after face-to-face activities resume. Although there is limited evidence for remote interventions with adolescents [37], a previous intervention involving adults identified significant positive improvements in MVPA and intrinsic motivation [38]. Schools are an obvious site from which to base youth PA interventions, however several issues are commonly cited when working in the school environment, such as timetabling constraints [39], inconsistencies in intervention implementation [40], varied availability of equipment and facilities [41], and lack of teacher adoption [42]. HERizon attempted to overcome such barriers as the intervention arms were not bound to a set location or weekly time slot. Instead, it gave participants the freedom to choose the type of physical activities that suited their schedule, interests, and available space/equipment, and provided support through informational and encouraging communications. These facilitators to implementation align with those presented in a Cochrane review, which concluded that the most effective PA interventions allowed participants to choose the type of PA they participate in and used phone calls to provide participants with feedback and support [43]. Irrespective of how the girls’ chose to be physically active, or if they decided to avail of additional support (e.g., live workouts and the Instagram group), all participants were encouraged to set goals and develop action plans and coping strategies during the programme. Similar to a previous behaviour intervention [44], these strategies were perceived as being integral to improving the girls’ PA. Although intervention dose and content should be standardised between participants for fair evaluation, in line with previous studies, flexibility and adaptation, through participant autonomy, were identified as being vital for intervention adherence and satisfaction, without compromising the intervention’s purpose [41,45].

Social interaction has long been recognised as an important facilitator to girls’ engagement in PA [46,47,48,49], with peer relationships having a significant positive association with adolescents’ motivation and quality of life [50,51]. HERizon provided participants opportunities for social interaction through Activity Mentor calls, live workouts, and a private Instagram group chat. These opportunities to connect with others on the programme were deemed especially important during the period of implementation, as the majority of participants were at home under strict COVID-19 lockdown restrictions. In a recent study, it was found that connecting with friends online during the pandemic reduced feelings of loneliness [52]. Further, it has been shown that social media can have a positive impact on health behaviours, such as improved PA and body composition [53,54]. Instagram was the chosen platform based on previous feedback from adolescent girls [7], and during focus groups participants confirmed that this was the most appropriate platform for the HERizon community as it was the social media they used most frequently (with Tik Tok and Discord being other recommended alternatives). Similar to Hutton and Robson [55], participant exit survey responses demonstrate moderate satisfaction and perceived usefulness of the group chat, however during focus groups and interviews it was evident that the majority of girls did not use the chat as intended. Many girls reported feeling “awkward” or uncomfortable putting messages in the chat and therefore it was predominantly the researcher who wrote into the group, e.g., reminding girls of upcoming live workouts. Much of the feedback received suggested that future iterations of HERizon should provide more opportunities for participants to introduce themselves and get to know one another better. Through researcher-facilitated group discussions, participants can begin to take ownership of the conversation and use the chat as a way to share interests and ideas [56]. However, it is acknowledged that building authentic, trusting relationships requires much time and effort even in ‘normal’ face-to-face interactions [57], and considerably more so in a remote setting [58]. Therefore, it is possible that a remote 12-week intervention provides insufficient time to foster a genuine sense of community.

### Strengths and Limitations

The comprehensiveness of our mixed-methods data provides detailed information on the reach, fidelity, adherence, acceptability, and context regarding the implementation of a remote PA intervention for adolescent girls. Triangulating information from several data sources allowed for several process evaluation questions to be investigated. Furthermore, this framework guided process evaluation was completed prior to analysing outcome measurements. However, a number of limitations are recognised within this study. Although a no-treatment control group allows for the evaluation and comparison of intervention components between groups, this design may be suboptimal as it does not account for external factors nor participant expectancies [59]. Further, non-treatment control groups may be unethical given the well documented benefits of regular PA for adolescents. Future PA interventions should consider a wait-list controlled trial. Not all participants (60.3%) returned completed exit surveys, thus potentially biasing our results as participants with strong views may have been more likely to complete the form [60]. Moreover, participant feedback on the intervention was only collected at the end of the programme, rather than throughout [21]. Finally, the first author of this process evaluation was also involved in intervention delivery, which may be a source of bias. To minimise bias, data were collected from participant and Activity Mentor logbooks, focus group participants were selected randomly, and the return of participant exit surveys was not influenced by the research team.

## 5. Conclusions

This process evaluation set out to gain insight into the reach and recruitment, delivery and receipt fidelity, adherence, acceptability, mechanisms of impact, and context of a 12-week remote PA intervention for adolescent girls. Findings suggest a successful recruitment strategy, as the target audience of adolescent girls from the UK and Ireland were enrolled into the study. There was a high level of fidelity as the majority of intervention components were delivered and received as intended. Participants in all intervention arms had good adherence to the PA protocol and participant satisfaction was high, however improvements can be made for the online group component of the intervention. Routine, sense of accomplishment, and accountability were identified as key mechanisms of impact within the intervention, and contextual factors, such as school holidays and exams were noted as having an influence on intervention implementation.

Based on this study’s results, the following recommendations are made to advance the quality of future evaluations:

- Context and its impact should not be undervalued when implementing a PA intervention in a real-world setting. Consideration should be given during intervention development to school terms, examination points, and typical vacation periods.
- Paid advertisements on social media that emphasise the accountability and community aspects of the intervention should be considered when recruiting adolescent girls.
- To foster a sense of community and belonging, providers should facilitate and encourage group discussion, e.g., ice-breaker tasks.
- Encouraging participant autonomy through choice and a flexible treatment design may increase long-term behaviour change and therefore should also be considered.

## Figures and Tables

**Table 1:** Details of intervention components and corresponding intervention arm.

Intervention Component	Description	PA ^1^ Programme Group	Behaviour Change Support Group	Combined Group	Comparison Group
PA ^1^ Logbook	This is a 25-page booklet that contains suggested PA ^1^ options and weekly optional worksheets to assist participants in setting goals and monitoring their progress. Participants were asked to record their weekly PA ^1^ in these logbooks.	X	X	X	X
Behaviour change support calls	Videocalls occurred on weeks 0, 1–6, 9, and 12 from their allocated Activity Mentor. Calls were based on a pre-planned session guide and aimed to support participants in becoming more physically active.		X	X	
Live group workouts	These sessions occurred twice per week for the duration of the intervention via an online video-conferencing software. Workouts were approximately 40 min and included a range of cardiovascular and resistance-based exercises.	X		X	
Text messaging	Using an online text messaging software, three standardised non-reply text messages were sent per week for the duration of the intervention. Messages provided reminders to live workouts, encouragement, and support.	X		X	
Private Instagram group chat	There were two Instagram groups, one for the PA ^1^ programme group and one for the combined group. The aim was to provide an opportunity for participants to interact with others in their group. The chat was moderated by a researcher and any messages that were sent by the researcher were replicated in both groups.	X		X	

^1^ PA physical activity.

**Table 2:** Process evaluation definitions and the components used to address research questions.

Process Evaluation Component	Definition and Research Question	Data Source
Reach and Recruitment	The degree to which the intended audience participates in the intervention, including maintenance of participants involvement in the intervention [28]. The procedures used to approach and attract participants. Did the intervention reach its target population? What procedures were used to recruit adolescent girls to the intervention, and which were most effective? What explains the decline in participation throughout the intervention?	# who expressed interest, # who consented, # who were eligible. Demographic and outcome measures compared to census data. Dropout rates and reasons. Focus groups and interviews.
Delivery fidelity	The degree to which intervention deliverers implement the intervention as intended by the intervention developers [26]. Was the intervention delivered as intended?	Mentor logbook intervention manual live workouts (frequency, content), Instagram group (frequency, content), text messages (total, frequency). Number logbooks sent to participants.
Participant receipt, engagement, and enactment	The degree to which participants’ understand, and apply the intervention principles [23]. How responsive were participants to the intervention?	Focus groups and interviews exit survey.
Adherence	A participant’s compliance with an intervention’s prescribed treatment [23]. What percentage of participants completed three PA sessions each week for 12 weeks according to their PA logbook, and did this percentage change depending on the intervention arm?	PA logbook focus groups and interviews, exit survey, mentor logbook.
Acceptability	The degree to which participants consider the intervention to be appropriate, based on anticipated or experiential cognitive and emotional responses to the intervention [29]. To what extent was the intervention appropriate for participants?	Focus groups and interviews, exit survey.
Mechanisms of impact	Participant responses to and interaction with the intervention, mediators and unexpected pathways and consequences [21]. What factors lead to positive/negative intervention effectiveness?	Focus groups and interviews, exit survey.
Context	Any aspect of the environment that may influence intervention implementation or study outcomes [21]. What were the external factors that affected the implementation of the intervention?	Focus groups and interviews, exit survey.

**Table 3:** Data collected and response rates.

Evaluation Method	Process Evaluation Component	Data Collection Time Frame	Number Completed	Response Rate
Exit survey	Fidelity of receipt, enactment fidelity, adherence, acceptability, mechanisms of impact, context	Post intervention	*n* = 91	60.3% of 151 baseline participants
Focus groups and individual interviews	Recruitment, receipt fidelity, adherence, acceptability, mechanisms of impact, context	Post intervention	*n* = 34 (11 focus groups & 3 interviews)	22.5% of 151 baseline participants
Mentor logbooks	Fidelity of study design, delivery fidelity, receipt fidelity, adherence	From intervention start to end (12 weeks)	*n* = 12	100%
PA logbook	Receipt of fidelity, enactment of fidelity, adherence	From intervention start to end (12 weeks)	*n* = 107	70.9% of 151 baseline participants

**Table 4:** Descriptive data for participants.

Characteristics	PA Programme Group (*n* = 36)	Behaviour Change Support Group (*n* = 44)	Combined Group (*n* = 34)	Comparison Group (*n* = 40)
Age, mean (SD), years	15.3 (1.0)	14.6 (1.3)	14.9 (1.1)	14.9 (1.2)
Reside in UK ^1^, *n* (%)	21 (58%)	20 (46%)	19 (56%)	15 (38%)
Ethnicity, *n* (%)				
*White*	29 (81%)	34 (77%)	28 (82%)	31 (78%)
*Asian or Asian British/Irish*	4 (11%)	3 (7%)	3 (9%)	5 (13%)
*African/Black*	1 (3%)	3 (7%)	1 (3%)	1 (3%)
*Mixed ethnic groups*	2 (5%)	2 (5%)	1 (3%)	1 (3%)
*Caribbean or Black British/Irish*	0	1 (3%)	1 (3%)	0
Socioeconomic status, *n* (%) ^a^				
*Tertile 1*	10 (28%)	9 (20%)	9 (26%)	18 (45%)
*Tertile 2*	20 (56%)	25 (45%)	23 (68%)	15 (38%)
*Tertile 3*	5 14%)	9 (20%)	2 (6%)	4 (10%)
PA, mean (SD), days	2.1 (1.5)	2.3 (1.6)	2.5 (1.6)	2.3 (1.9)

^1^ UK United Kingdom, PA physical activity. ^a^ Socioeconomic status was determined based on home postcode using the Irish Pobal HP Deprivation Index and the UK Index of Multiple Deprivations (1 = most deprived, 2 = median deprived, 3 = least deprived).

## Supplementary Materials

The following are available online at https://www.mdpi.com/article/10.3390/ijerph19020966/s1, Figure S1. Sample pages of the PA logbook; Table S1. Overview of 12-week delivery framework for Activity Mentor behaviour change support calls; Table S2. Postintervention exit survey; Table S3. Interview guide of sample questions asked during semi-structured interviews.
