# Supplementary material for: What Happened in ‘The HERizon Project’?—Process Evaluation of a Multi-Arm Remote Physical Activity Intervention for Adolescent Girls
Source: Int J Environ Res Public Health. 2022 Jan 15;19(2):966. doi: 10.3390/ijerph19020966 (PMC8775378; doi:10.3390/ijerph19020966)
Supplement: Supplementary file 1 [file ijerph-19-00966-s001.zip › ijerph-1447153-supplementary.pdf]

Supplementary material

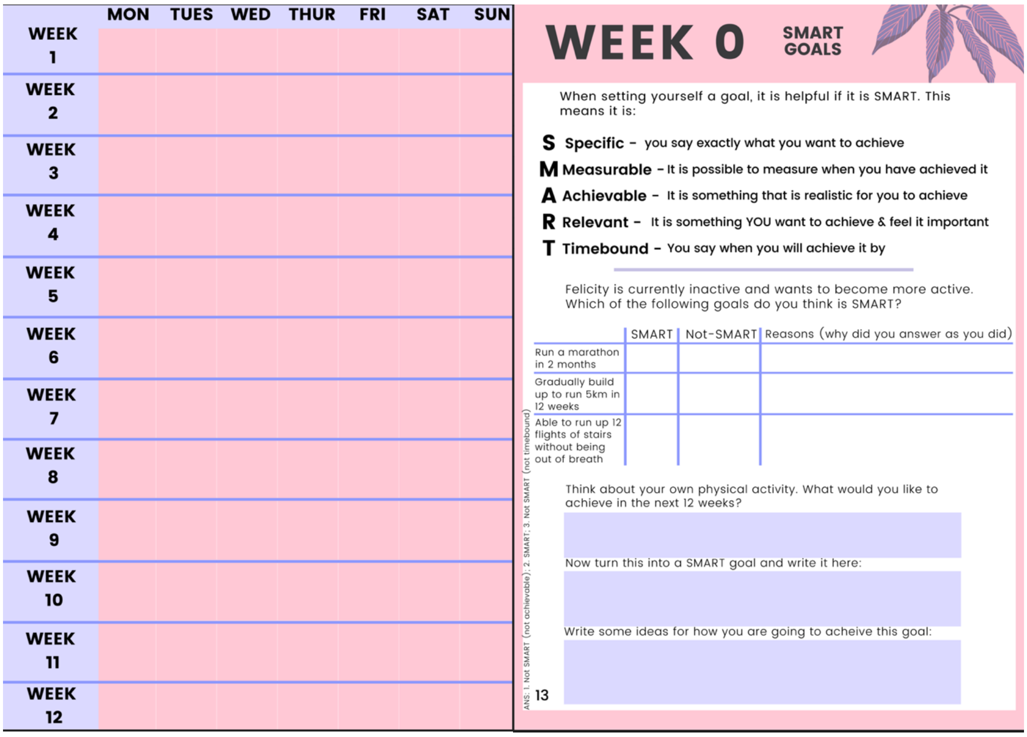

Figure S1. Sample pages of the PA logbook.

Table S1. Overview of 12-week delivery framework for Activity Mentor behaviour change support calls.

	Description of session	
Week 0 (30 mins)	Intro - rapport building and goal setting	
Week 1	Setting action plans	
Week 2	Barrier identification	Review/adjust action plan
Week 3	*Action plan review (no specific topic)*	
Week 4	*Action plan review (no specific topic)*	
Week 5	Coping planning	
Week 6	Reflect on achievements	
Week 9	Coping planning	
Week 12 (30 mins)	Reflect on achievements<br>Coping planning	

Table S2. Postintervention exit survey.

FOR ALL PARTICIPANTS

1. What was your favourite part of the HERizon Project?
2. What was your least favourite part?
3. Please share with us any suggestions on ways we can improve the programme
4. Has the HERizon Project helped you improve your attitudes/behaviours towards physical activity? (1= no it seemed to make things worse, 5 = yes it helped a lot)
5. On a scale of 1-5 how competent do you feel to carry on with your physical activity after the programme has ended? (1 = not at all competent, 5 = very competent)
6. On a scale of 1-5 how confident do you feel you have the support around you (e.g. family, friends) to help you continue your physical activity after the programme? (1 = not at all confident, 5 = very confident)

7. On a scale of 1-5 how much choice and options do you feel you had during HERizon? (1 = no choice or options at all, 5 = loads of choice and options)
8. On a scale of 1-5, do you feel you understand the reasons for being physically active and their importance? (1= not at all, 5 = very much).
9. What level/tier of COVID-19 lockdown restrictions was your country in at the end of your 12-week programme? E.g. were shops open, could you travel, could you meet your friends, Were non-essential shops open? E.g. retail stores, Were restaurants/cafes open for dine in? Were you able to meet friends/family outside your "bubble" indoors? Were you able to travel outside your locality? If you know the date that the country moved out of full lockdown, please type it here:
10. During COVID-19 lockdown, did you have any virtual PE classes? If yes, How many days per week? How many minutes were each class? Can you please describe what you did in virtual PE classes?
11. Have you returned to school or are you still working from home?
12. If your school has reopened: Have your weekly commitments changed? More commitments, less commitments, same. If more, what are these extra commitments? Has your weekly routine/timetable changed? If so, how do you feel your behaviour/motivation towards physical activity has changed? More motivated, less motivated, the same. Can you explain why there has been a change?
13. Did you use your logbook to monitor your weekly physical activity sessions? Scale of 1-5 (1= not at all, 3= sometime, 5= all the time) If not, what did you use, if anything?
14. Did you use the example physical activities in the logbook? E.g. YouTube videos and IGTVs. Scale of 1-5 (1= not at all, 3= sometime, 5= all the time). If you used these examples, what ones did you like/ not like and why, If you did not use these examples, what activities did you do
15. Please use this space to make any further comments about the HERizon programme.
FOR GROUPS WITH ACTIVITY MENTORS (PLUS GENERAL QUESTIONS)
16. Who was your activity mentor?
17. How satisfied are you with the amount of help you received? Scale of 1-5 (1 = not satisfied, 5= very satisfied), How useful did you find the calls for helping you become more physically active (1 = not at all useful, 5 = very useful), How useful did you find the calls for helping you feel supported (1 = not at all useful, 5 = very useful), Are there any topics you wish had been covered in the sessions?
18. What did your mentor do well during the calls? Give at least 1 example, What could your mentor improve on for future calls? Give at least 1 example
19. How comfortable did you feel talking to your mentor? Scale of 1-5 (1= not comfortable at all, 5=very comfortable)
20. How much did you enjoy the calls? (1=not at all, 5= very much), Why did you enjoy them/ not enjoy them?
21. Do you feel your mentor was knowledgeable about the content of the sessions? (1 = not at all, 5 = very much)
22. What did you think of the length of the calls? Too long, too short, just right.
23. If you were to do the programme again, would you want a mentor again? Yes, no, unsure. Please explain your response.
24. If a friend wanted support in becoming more active, would you recommend her to join a HERizon programme? (1= no definitely not, 5= yes definitely).
25. Would you like to make any further comments about your mentor?
FOR GROUPS WITH LIVE WORKOUT/SOCIAL MEDIA/ TEXTS (PLUS GENERAL QUESTIONS)
26. Did you take part in any live workouts? If yes, on a scale of 1-5 how much did you enjoy them? (1= not at all, 5= really enjoyed them), What was your favourite aspect of the classes? What was your least favourite? Did you ever join a class with a friend/ family member? If no, what stopped you from trying a live workout?
27. Did you watch back on any of the recorded live workouts on the Google Doc folder? Is there another platform that you would prefer for these recorded workouts to be uploaded to?
28. Is there anything you recommend changing about the live workouts?
29. Did you join the Instagram private group? If yes, On a scale of 1-5 how much did you enjoy being part of this group? (1= not at all, 5= really enjoyed them). On a scale of 1-5 how useful was it being part of this group? (1=not useful at all, 5=really useful). If no, what stopped you from joining this group? Do you have any suggestions on other ways we could use social media to create a HERizon community?
30. Did you receive 3 weekly text messages from the HERizon Project? If yes, on a scale of 1-5 please rate how useful these messages were (1=not useful at all, 3=somewhat useful, 5=really useful). Was the frequency of these messages acceptable? Too frequent, not enough, just right. Is there anything you recommend changing about the text messages?

Table S3. Interview guide of sample questions asked during semi-structured interviews.

Can you tell me anything you have liked about the programme so far?
Can you tell me anything you don't like?
What do you think would increase the chances of you sticking to the programme? I.e. doing your 3 exercise sessions per week.
Tell me what you think of the weekly texts and calls
How could we make this programme more enjoyable for you? Could you give me your opinion and feedback?
Tell me your thoughts on the assessments? I.e. questionnaires and fitness tests.
